# Conformational Changes and ATP Hydrolysis in Zika Helicase: The Molecular Basis of a Biomolecular Motor Unveiled by Multiscale Simulations

**DOI:** 10.1021/jacs.3c09015

**Published:** 2023-11-03

**Authors:** Adrián García-Martínez, Kirill Zinovjev, José Javier Ruiz-Pernía, Iñaki Tuñón

**Affiliations:** Departamento de Química Física, Universidad de Valencia, 46100 Bujassot, Spain

## Abstract

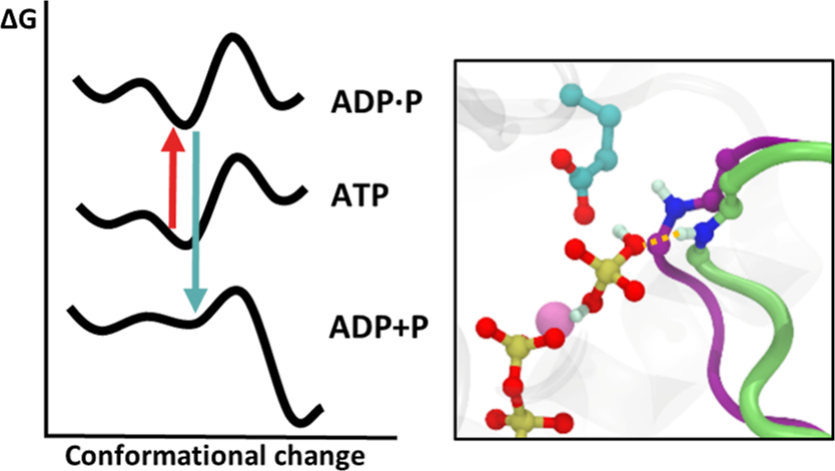

We computationally study the Zika NS3 helicase, a biological motor, using ATP hydrolysis energy for nucleic acid remodeling. Through molecular mechanics and hybrid quantum mechanics/molecular mechanics simulations, we explore the conformational landscape of motif V, a conserved loop connecting the active sites for ATP hydrolysis and nucleic acid binding. ATP hydrolysis, initiated by a meta-phosphate group formation, involves the nucleophilic attack of a water molecule activated by Glu286 proton abstraction. Motif V hydrogen bonds to this water via the Gly415 backbone NH group, assisting hydrolysis. Posthydrolysis, free energy is released when the inorganic phosphate moves away from the coordination shell of the magnesium ion, inducing a significant shift in the conformational landscape of motif V to establish a hydrogen bond between the Gly415 NH group and Glu285. According to our simulations, the Zika NS3 helicase acts as a ratchet biological motor with motif V transitions steered by Gly415’s γ-phosphate sensing in the ATPase site.

## 1 Introduction

Biomolecular motors, proteins facilitating directed motion, are essential for cellular functions like intracellular transport and replication of genetic material.^1−5^ These motors convert chemical energy into conformational changes, typically through ATP hydrolysis.^1^ While the ATP hydrolysis mechanism in biological motors has been extensively studied and catalytic strategies shared by different enzymes have been identified,^6^ the atomistic details for the transduction of the reaction free energy into mechanical activity remain unknown in most cases.^7−10^ Two main mechanisms are usually discussed in connection with free energy consumption and the generation of directed motion in biomolecular motors: power strokes and Brownian ratchets. A power stroke involves a free-energy-releasing conformational change driven by strain or stored elastic energy following binding, reaction, or product release of a chemical fuel. Alternatively, in a Brownian ratchet mechanism, the conformational change in the forward direction occurs due to random thermal forces. However, the motion is directed because chemical changes prevent the system from evolving in the backward direction. Arguments based on microscopic reversibility and the prevalence of low Reynolds number conditions operating inside cells^11,12^ seem to favor a mechanism based on Brownian motion as more plausible for biomolecular motors.

Helicases enzymes, a biomolecular motor family, use ATP hydrolysis to unwind nucleic acid double-strands, a pivotal process in cell replication. As the helicase performs its ATPase activity, the nucleic-acid-binding site undergoes a conformational change that modulates the intensity of the interaction with the nucleic acid: it is tighter in the nucleotide-free state and weaker in the presence of ATP.^13^ Thereby, the ATPase activity may promote the translocation of the helicase along the nucleic acid strand. One prominent example of this enzymatic family is the NS3 helicase of the Zika virus (or ZIKV-NS3hel). The NS3 helicase belongs to the second superfamily (SF2) of helicases^5^ and displays ATPase-dependent helicase activity. NS3hel is responsible for the unwinding of a double-stranded RNA replication intermediate and for translocation along single-stranded RNA, a critical activity for the replication cycle of the virus, making it a potential antiviral target.

**Figure 1:**
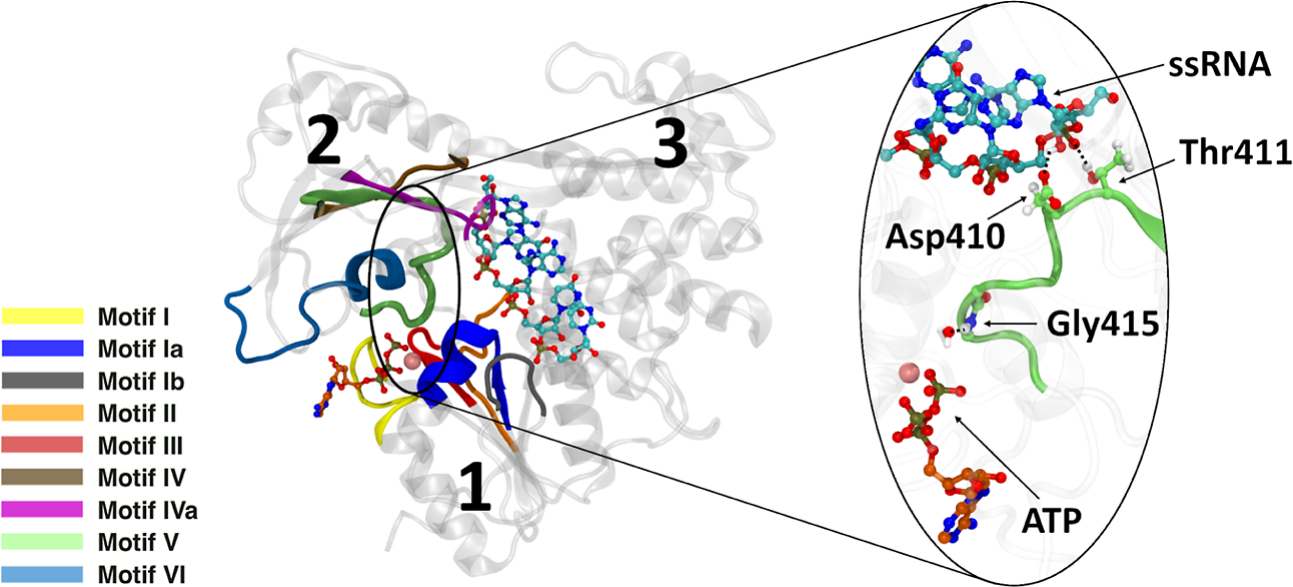
Zika virus NS3 helicase structure (a combination of PDB structures 5GJB and 6S0J). Subdomains are labeled as 1, 2, and 3. The ATPase active site is located between subdomains 1 and 2, and the RNA binding cleft is located at the center between the three subdomains. The conserved structural motifs are represented in different colors. Motif V is highlighted in light green as a possible communication channel among the ATPase active site and the RNA binding cleft due to the presence of residues interacting with both ligands.

The NS3 helicase structure is depicted in Figure 1. It features three subdomains, with the ATP binding pocket situated between the first two and the RNA binding cleft placed in the center of the three. Around the two active sites, there are several conserved motifs, critical for ATP binding, interdomain communication, and RNA binding. Recently, ZIKV-NS3hel was crystallized with a transition state analogue (TSA) of ATP hydrolysis.^14^ Structural analysis revealed two different conformations for motif V, one of the conserved sequences that connects the ATPase and RNase activities in NS3hel.^15,16^ On the side of the RNA binding site, motif V contains a highly conserved threonine residue (Thr411) that forms a direct hydrogen bond contact with the nucleic acid.^17^ On the ATPase site, the same motif displays a hydrogen bond interaction between the backbone amide group of Gly415 and a water molecule, which is a candidate to perform the ATP hydrolysis reaction.^14^ Consequently, motif V may act as a communication channel between the ATPase and RNAase activities of NS3hel, facilitating the conversion of chemical energy into directed motion through conformational changes coupled to the ATPase cycle.

These structural observations make ZV-NS3hel an interesting model to study the coupling between the chemical energy released during ATP hydrolysis and conformational changes. In this paper, we present the results of a series of molecular mechanics (MM) and hybrid quantum mechanics/molecular mechanics (QM/MM) simulations where we investigate the interplay between the conformational state of motif V and ATP hydrolysis. We will first explore the reaction free energy landscape for the ATPase activity of ZV-NS3hel, determining the most favorable reaction mechanism and its correlation with the conformational state of motif V. We will then show how, in turn, the conformational free energy landscape of this motif depends on the stage of the ATPase cycle, in particular on the presence of the γ-phosphate group of ATP in the ATPase active site. Interestingly, our simulations unveil that Gly415 acts as a molecular sensor for the presence of the γ-phosphate group, which can trigger the conformational change in motif V as a function of the presence or absence of this group. These insights advance our understanding of the linkage between ATP hydrolysis and motion in helicases and potentially other molecular motors.

## 2 Methodology

### 2.1 MM Molecular Dynamics Simulations

The Protein Data Bank structure 6S0J was selected to carry out the study. This structure corresponds to the monomeric form of the ZIKV NS3 helicase in complex with ADP-MgF_3_H_2_O,^14^ a complex that mimics the transition state (TS) of ATP hydrolysis. The PDB structure contains two different conformations for motif V, A and B, which were selected to prepare the two initial structures of the system. These structures correspond to the RNA-free state, which seems to be an advantage to explore the conformational diversity of motif V, as far as the presence of RNA is known to constrict the active site.^14^ It must be noticed that the ZIKV helicase displays intrinsic ATPase activity, with a *k*_cat_ = 1.18 s^–1^ (*T* = 298 K) in the absence of RNA.^18^ Furthermore, structure 6S0J seems to be in a catalysis-ready state for hydrolysis independently of RNA binding. The Glu286 residue is found in an optimal position to deprotonate the catalytic water (H_2_O_r_).

In order to prepare the simulations of the Michaelis complex for the ATPase activity, the MgF_3_H_2_O moiety of the substrate was replaced by a phosphate group, thus turning the substrate into ATP. This was achieved with Maestro 13.5 software preserving the coordination of the added phosphate group with the Mg^2+^ ion. Missing residues (from Thr246 to Thr255) were added by using AlphaFold^19^ under the ChimeraX 1.3 interface.^20^ The absent hydrogen atoms were added using the tleap tool from AmberTools18.^21^ This was done in accordance with the standard protonation states of amino acid residues at pH 7.4. The protonation state of all titratable residues can be obtained from the files provided (data availability statement). Parameters for ATP and ADP were taken from the work of Meagher et al.^22^ On the other hand, parameters for the negatively charged inorganic phosphate H_2_PO_4_^–^ were obtained with a nonstandard residue parametrization procedure using the Antechamber tool^23^ from AmberTools18. The partial charges of the phosphate group atoms were calculated using the restrained electrostatic potential method^24^ at the HF/6-31G* level as implemented in Gaussian16.^25^ Protein amino acids were described using the ff14SB force-field.^26^ The protein–substrate complex was solvated in a box of water molecules described with the TIP3P^27^ potential with a buffer region of at least 12 Å from protein/substrate atoms to the limits of the simulation box. The charge of the system was neutralized by the addition of one Cl^–^ ion. Long-range electrostatic interactions were described using the particle mesh Ewald method (PME) with the cutoff radius for short-ranged interactions set to 10 Å.

MM molecular dynamics (MD) simulations of the ZIKV NS3 helicase in the Michaelis complex with ATP were run starting from conformations A and B. The MD simulations followed a standard equilibration procedure consisting of four stages. The first stage was a minimization in four cycles using the steepest descent method for the first 200 steps followed by the conjugated gradient method until the root mean square of the gradient was below 10^–3^ kcal·mol^–1^·Å^–1^ or until the minimization algorithm could not find any configuration lower than the present one. A harmonic restraint of 200 kcal·mol^–1^·Å^–2^ was applied to different sets of atoms at each optimization step. The first step was carried out with every atom except hydrogens restrained; in the second step, water molecules were released, while amino acid side chains and protein backbone atoms were released in the third and fourth steps, respectively. In the second stage, the system was heated from 0 to 300 K using the Langevin thermostat with a linear heating rate of 2.1 K·ps^–1^ in the NTP ensemble using Berendsen’s barostat. The backbone atoms of the protein, heavy atoms of the substrate, and the divalent cation and its coordination sphere were restrained positionally using a harmonic potential with a force constant of 20 kcal·mol^–1^·Å^–2^. Such restraints were released during the equilibration stage from 15 to 3 kcal·mol^–1^·Å^–2^, decreasing 3 units every 1 ns. After 5 ns, the positional restraints were removed, and the equilibration was continued during 2 ns of additional NTP simulation. Then, to ensure enough sampling, six replicas of 1 μs each in the *NVT* ensemble were run. All simulations were carried out using the Amber18 GPU version of PMEMD.^28,29^ The time step was set to 2 fs using the SHAKE algorithm^30^ to treat bonds involving hydrogen atoms.

### 2.2 QM/MM Calculations

**Figure 2:**
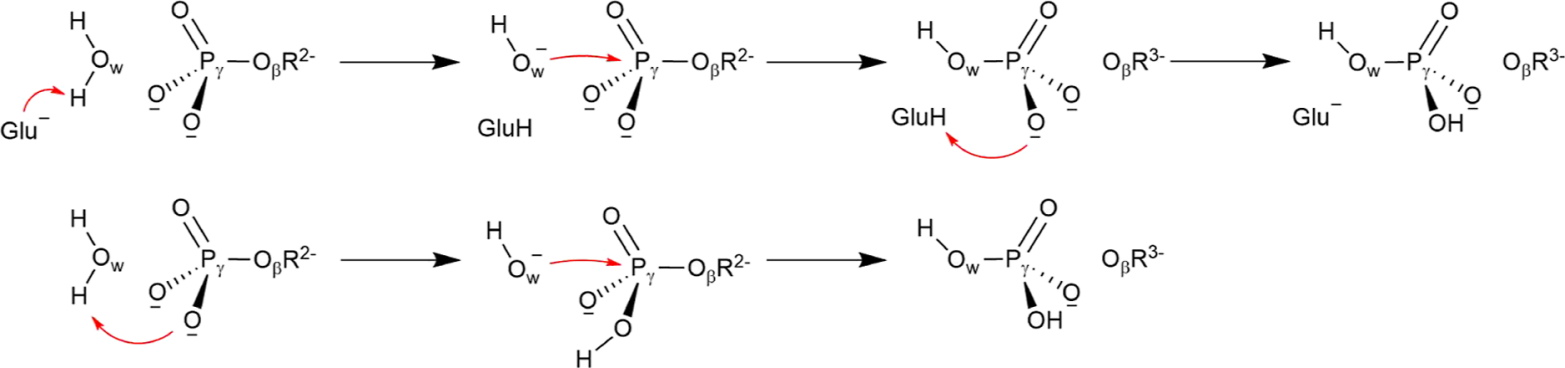
Scheme of ATP hydrolysis for a base-assisted mechanism (top) and a substrate-assisted mechanism (bottom).

Hybrid QM/MM simulations were used to describe the ATP hydrolysis reaction in the active site of the ZIKV NS3 helicase. The QM region was described using the B3LYP^31,32^ functional with the 6-31G(d) basis set including D3 dispersion corrections.^33^ This functional was chosen as a good compromise between computational efficiency and reliability for systems similar to those studied here^34^ and in particular for the hydrolysis of phosphate anions.^35^ Inclusion of dispersion corrections improves the calculation of reaction barriers in enzymes at a negligible computational cost.^36^ The QM region includes the triphosphate moiety of ATP, the reactive water molecule, the catalytic base Glu286, and the divalent anion Mg^2+^ and its coordination sphere (see Figure S1). QM/MM calculations were also carried out with Amber18 in combination with Gaussian16^25^ for density functional theory calculations. The cutoff radius for QM/MM interactions was set equal to 15 Å. Different mechanistic proposals, corresponding to a general base-assisted proton abstraction and a substrate-assisted proton abstraction^37^ from the catalytic water, were explored (see Figure 2). In order to accelerate the convergence of the B3LYPD3/MM explorations, we used as the initial guess simulations obtained at the DFTB3/MM level^38^ with the OPhyd^39^ parameters, a set of parameters developed to describe complex phosphorus chemistry with improved accuracy.

### 2.3 Free Energy Calculations

The free energy surfaces (FESs) associated with ATP hydrolysis and conformational changes were explored with the adaptive string method (ASM),^40^ an implementation of the string method developed in our research group. The ASM is used to determine the minimum free energy path (MFEP) on an FES spanned in a space defined by collective variables (CVs), which are the most important degrees of freedom driving the process. N string nodes, replicas of the system centered at different values of the CVs, are simulated, and their positions evolved according to the free energy gradient while being kept equidistant until the MFEP is found. Subsequently, a single path-CV (denoted as s) is defined and used as the reaction coordinate to compute the free energy profile with the umbrella sampling algorithm.^41^

For the study of ATP hydrolysis, the set of CVs is composed of the distances of those bonds being broken or formed during the chemical reaction together with the pyramidalization coordinate of the P_γ_ atom. The set of CVs for each explored reaction mechanism is detailed in the Results section. The strings contained 70 to 80 nodes depending on the mechanistic proposal. The mass of the transferred hydrogen atoms was changed to that of deuterium, which allowed the use of a time step of 1 fs. The string was propagated at the QM/MM level until the root-mean-square deviation (rmsd) of the CVs was below 0.1 amu^1/2^ Å for 1.5 to 2 ps. The simulations then enter the umbrella sampling stage along the path-CV coordinate to obtain the sampling required to compute the free energy profile. These simulations were run for at least 10 ps and then integrated using the weighted histogram analysis method (WHAM).^42,43^

For the study of the conformational change of motif V, the chosen CVs are the main backbone torsional angles that change between conformations and are detailed in the Results section. To be able to sample correctly conformational changes, the simulations were run at the MM level using GPU-accelerated MD simulations with a time step of 2 fs. The strings contained 48 nodes and were propagated until the rmsd was below 0.15 amu^1/2^ Å for 1 to 1.5 ns. Once the MFEP was converged and the path-CV defined, umbrella sampling along the path-CV was carried out for at least 7.5 ns. The WHAM was also employed to obtain the free energy profile.

## 3 Results

### 3.1 Motif V Conformations and ATPase Active Site

In order to explore the conformational landscape of motif V in the ATP–helicase complex, we analyzed the probability distributions of the ψ_*i*_ and φ_*i*+1_ backbone dihedral angles for six replicas of 1 μs-long MM MD simulations starting from different initial configurations obtained from the 6S0J PDB structure (see the Methodology section). For reference, the dihedral angles corresponding to the two conformations observed in the X-ray structure are given in Table S1. These two conformations differ mainly in the values of ψ_414_ and φ_415_. Since these angles precede and follow the location of the backbone NH group of Gly415, they are likely to strongly affect its orientation.

**Figure 3:**
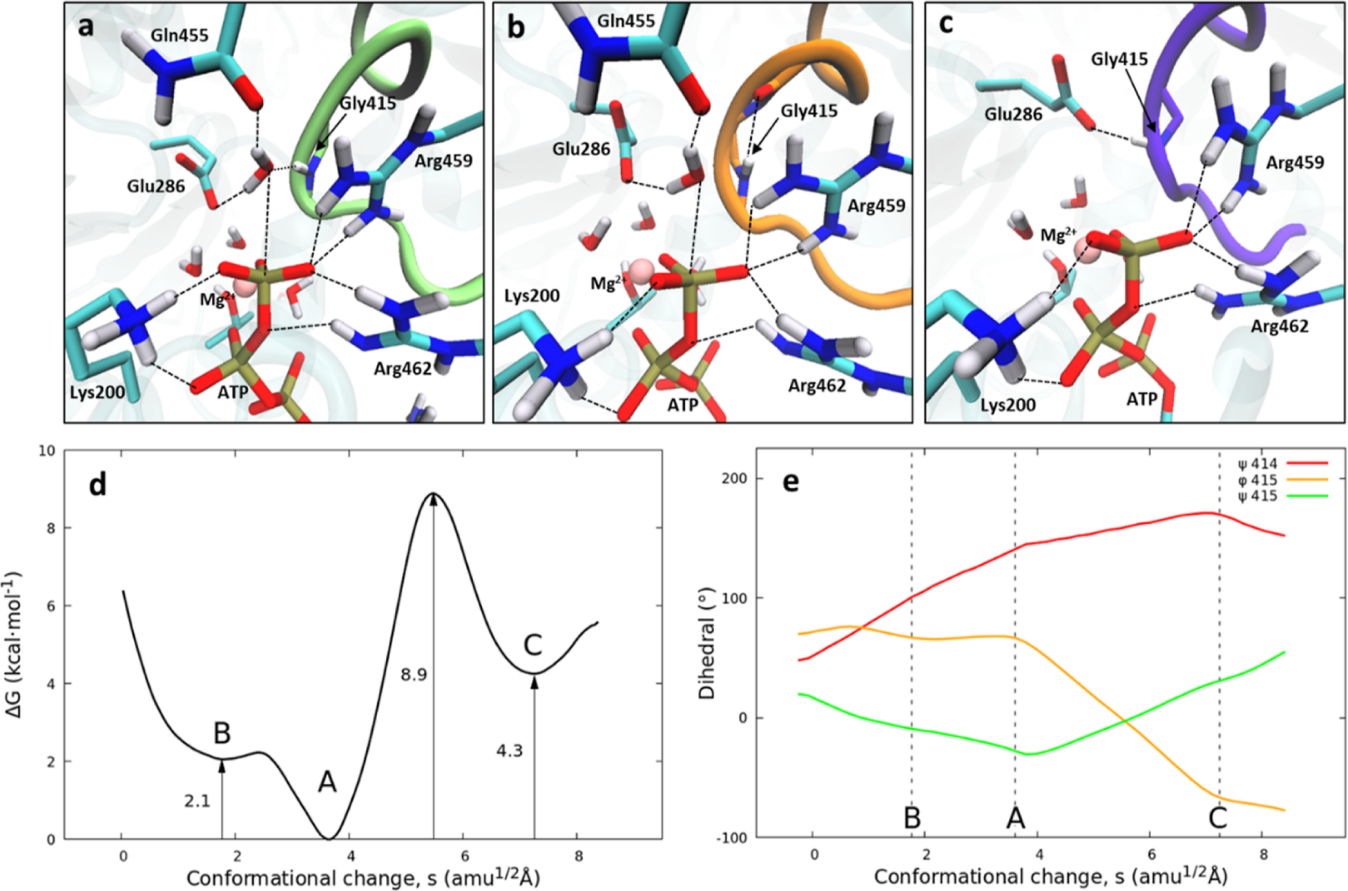
Conformational landscape associated with the ψ_414_/φ_415_ torsional angles of motif V in the ZIKV-NS3 helicase obtained from MM MD simulations. (a) Enzyme–substrate complex in conformation A of motif V (green). (b) Enzyme–substrate complex in conformation B of motif V (orange). (c) Enzyme–substrate complex in conformation C of motif V (purple). (d) Free energy profile along the path-CV (s) for the B → A → C transition. (e) Evolution of the CVs employed to study the conformational transition of the motif V transition along the MFEP (ψ_414_, φ_415_, and ψ_415_ backbone torsional angles).

The results of this analysis are shown in Figure S2 as a heatmap representation of the ψ_*i*_ and φ_*i*+1_ joint distribution. According to these results, the configurational space of dihedral pairs from ψ_405_/φ_406_ to ψ_412_/φ_413_ is confined to a single basin throughout all of the replicas. The loop region spanned by residues 405 to 413 goes through the contact region among the three subdomains and into the RNA binding cleft and thus is expected to have a tight configuration. In contrast, the loop region defined by residues 414 to 417 is close to the ATPase active site and is solvent exposed, which leads to a larger flexibility. Analysis of the ψ_414_/φ_415_ pair (see Figure S2) shows that motif V can be found in at least three different conformations. These dihedral angles are those that discriminate between conformations A and B in the X-ray structures, presenting ψ_414_/φ_415_ values of 145°/65° and 58°/92°, respectively (see Table S1). Moreover, a new conformation appears, which from now on will be referred to as conformation C. This conformation is centered around ψ_414_/φ_415_ values of 160°/–74°. As seen in Figure S2, conformations A–C also show slight variations in the values of ψ_415_. Figure 3 depicts representative structures of the Michaelis Complex in the three sampled conformations of motif V. The three conformations present a very similar pattern of enzyme–ATP interactions. Specifically, Arg459 interacts with the γ-phosphate group, while Arg462 and Lys200 interact with both γ and β phosphates. All of them are highly conserved residues across the viral DExH-like SF2 subfamily.^44^ Similarly, the reactive water molecule (H_2_O_r_) interacts with Glu286 and Gln455, acting as a H-bond donor to both residues. As expected, motif V conformations differ in the orientation of the peptide group between residues 414 and 415. In conformation B, the backbone NH group of Gly415 forms an intraloop hydrogen bond with the carbonyl oxygen atom of Met414. In the transition from B to A, the loop unfolds, and the NH group of Gly415 directly points to the ATPase active site, forming a hydrogen bond with H_2_O_r_, placed close to the γ-phosphate group. Finally, in conformation C, the backbone NH group of Gly415 forms a hydrogen bond with the carboxylate group of Glu286. This observation is of particular interest because this residue is expected to abstract the proton of the catalytic water, suggesting that conformation C could not be conducive for catalysis.

To properly quantify the free energy changes associated with the conformational landscape of motif V, we used the ASM (see the Methodology section) to trace the MFEP connecting the three conformations at the MM level. The CVs used for this purpose are the backbone torsional angles that differentiate the three conformations: ψ_414_, φ_415_, and (to a minor extent) ψ_415_. The resulting free energy profile and the evolution of the CVs are shown in Figure 3d,e. The free energy profile clearly displays three minima corresponding to conformations B, A, and C (from left to right). A is characterized as the most stable conformation for motif V in the Michaelis complex, while conformations B and C are found 2.1 and 4.3 kcal·mol^–1^ above the ground state, respectively. The barrier between conformations B and A is small, which explains the frequent transitions observed between them in Figure S2. Instead, conformation C is separated by a significant free energy barrier from conformation A, 8.6 kcal·mol^–1^. The evolution among these three conformations is determined by the rotation of the Met414-Gly415 peptide group, governed by the increase of ψ_414_ and the decrease of φ_415_ (see Figure 3e).

### 3.2 ATP Hydrolysis Mechanism in the Zika NS3 Helicase

**Figure 4:**
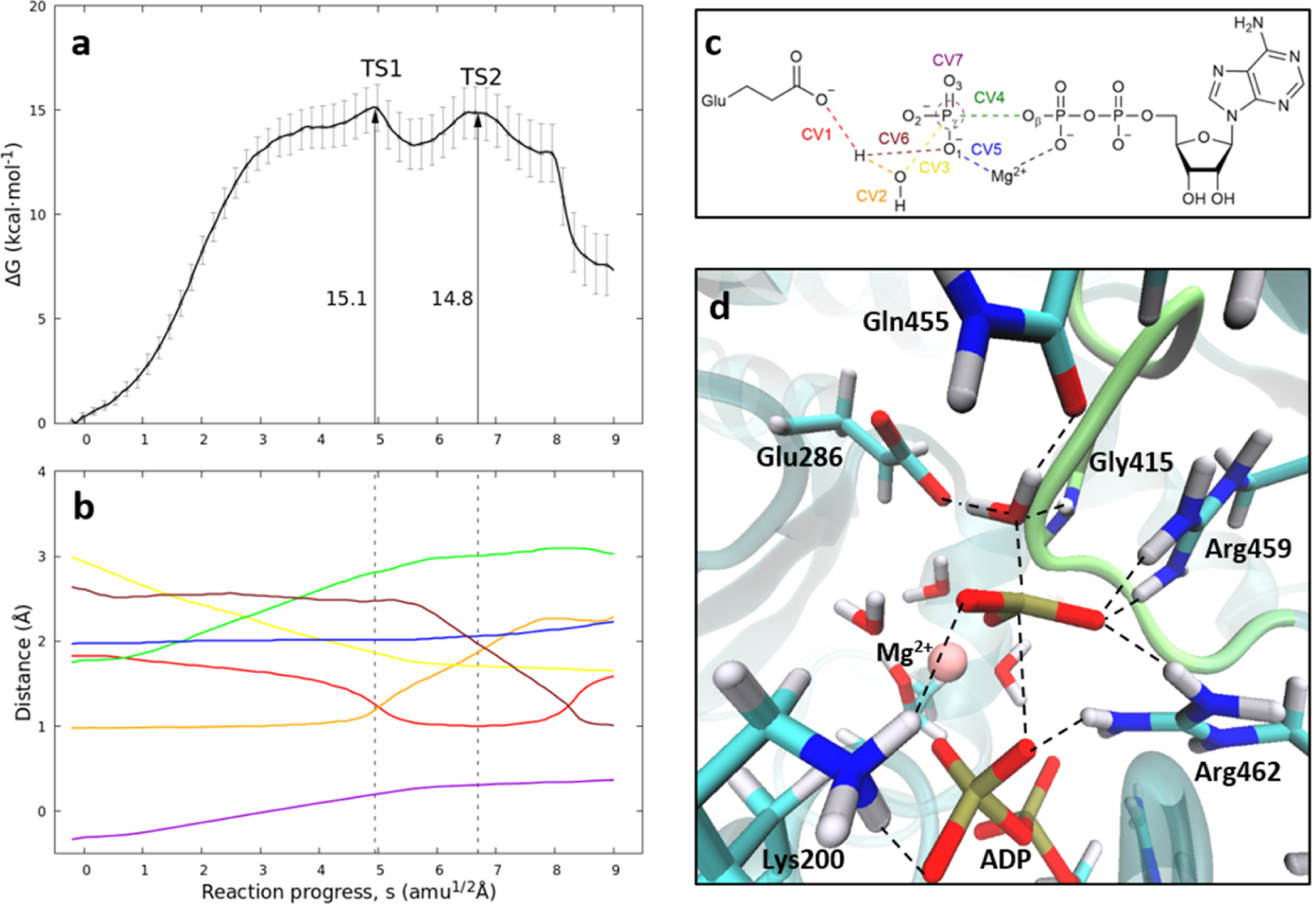
Base-assisted mechanism for the ATP hydrolysis in the ZIKV-NS3 helicase in conformation A of motif V. (a) B3LYPD3/6-31G*/MM free energy profile along the path-CV (s) for ATP hydrolysis up to the formation of ADP and dihydrogen phosphate. (b) Evolution of the CVs along the path-CV. (c) CVs employed to study the base-assisted reaction mechanism: distances H-OE:Glu286 and H–O_w_ (CV1 and CV2) for the deprotonation of the water molecule, O_r_–P_γ_ and O_β_–P_γ_ (CV3 and CV4) for the nucleophilic attack and cleavage of the phosphoanhydride bond, and O1/ATP-Mg^2+^ (CV5) for the coordination of γ-phosphate to the divalent ion, H–O1/ATP for the second proton transfer to form H_2_PO_4_^–^ (CV6). We also included the plane inversion of P_γ_ (CV7), measured as the distance between P_γ_ and the plane defined by the three bonded oxygen atoms. (d) Structure of the rate-limiting step (TS1). Motif V is depicted in lime color.

The free energy landscape for the ATP hydrolysis mechanism was explored by starting from the Michaelis complex of the ZIKV NS3 helicase with ATP at the B3LYPD3/MM level. Conformation A was selected as the most promising one for catalysis because of the hydrogen bond interaction between the backbone NH group of Gly415 and the catalytic water placed close to the γ-phosphate group. This hydrogen bond interaction facilitates the orientation of the water molecule for nucleophilic attack and increases its acidic character. Results of the preliminary DFTB3/MM exploration are presented in Figure S3. The resulting B3LYPD3/MM free energy profile and evolution of the CVs that define the chemical transformation are shown in Figure 4a,b. The selected CVs are detailed in Figure 4c.

According to the evolution of the CVs along the MFEP shown in Figure 4b, the reaction path converged toward a sequential mechanism. The first step is the dissociation of the O_β_-P_γ_ bond (CV4) leading to the formation of a planar meta-phosphate group. The formation of the meta-phosphate is favored by positively charged residues Lys200, Arg459, and Arg462, which stabilize the departure of this group through electrostatic interactions. The formation of the planar meta-phosphate group is followed by the nucleophilic attack of the water molecule (CV3 is reduced up to 1.8 Å at TS1, shown in Figure 4d) and its deprotonation by Glu286 (CV1 and CV2 that cross at TS1, with distances equal to 1.2 Å, as observed in Figure 4b). As observed, TS1 is the rate-limiting step of the ATP hydrolysis reaction, with an associated free energy barrier of 15.1 kcal·mol^–1^. This value is in good agreement with the derived from the experimental rate constant,^18^ 17.3 kcal·mol^–1^. After TS1, a high energy intermediate is reached, which corresponds to protonated Glu286 with HPO_4_^2–^ in the active site. The next step consists in a second proton transfer from Glu286 to the inorganic phosphate, which requires a rearrangement of hydrogen bonds through a second transition state, TS2. After this TS, the proton is directly transferred from Glu286 to the inorganic phosphate (see the crossing point of CV1 and CV7 after TS2), leading to the formation of H_2_PO_4_^–^. This proton is transferred to the oxygen atom of the phosphate group coordinated to the divalent ion, weakening the interaction in the coordination sphere and slightly lengthening the O1(Pho)–Mg^2+^ distance (see CV5). The ATP hydrolysis process results in the formation of ADP and H_2_PO_4_^–^, displaying a reaction free energy of +7.6 kcal·mol^–1^. As discussed below, several possible rearrangements might occur after ATP hydrolysis that can lower the free energy.

The free energy landscape of the base-assisted mechanism was also explored with conformation B of motif V, using the same active space of CVs (Figures S4 and S5). The reaction path of ATP hydrolysis converged to the same mechanism as that in conformation A, with a higher activation free energy of 18.9 kcal·mol^–1^. We attribute the larger activation free energy to the lack of a hydrogen bond between motif V and the nucleophilic water that decreases the acidic character of the hydrolytic water molecule, increasing the free energy cost associated with the proton transfer to Glu286. Our results confirm that conformation A is more favorable than conformation B to catalyze the base-assisted mechanism for ATP hydrolysis. As explained before, conformation C was discarded because of the hydrogen bond interaction established between the backbone NH group of Gly415 and Glu286 that decreases its basicity.

We also explored a substrate-assisted mechanism for ATP hydrolysis in conformation A (Figure S6) in which the proton of the nucleophilic water is directly transferred to the γ-phosphate group (see Figure 2 and the Methodology section). The reaction path of this mechanism converged to a sequential dissociative mechanism with a very high free energy barrier, 41.7 kcal·mol^–1^, confirming that the substrate-assisted path is not feasible. Note that the base-assisted mechanism, clearly more favorable than the substrate-assisted one, requires that residue Glu286 be in its basic form. The p*K*_a_ value of this residue is very low because of the vicinity of the magnesium ion (PropKa^45,46^ predicts a negative value), and thus, the base-assisted mechanism could be available at a wide range of pH values. Studies on the NS3 helicase from HCV, another member of the helicase SF2 superfamily, confirm that the rate of ATP turnover in the absence of RNA does not change appreciably between pH values of 6 and 10. Instead, binding of the nucleic acid is clearly favored, lowering the pH,^47^ which could be due to changes in the protonation state of residues close to the nucleic acid binding site.

### 3.3 Free Energy Release after ATP Hydrolysis

The ATP hydrolysis mechanism described above corresponds to an endergonic process in which the products are ADP^3–^ and the dihydrogen phosphate (H_2_PO_4_^–^) coordinated to the Mg^2+^ ion through one of the protonated oxygen atoms. Free energy can be released because of chemical or nonchemical rearrangements occurring after the ATP hydrolysis.

A possible strategy to diminish the free energy is proton tautomerism. We explored possible proton transfer events taking place between inorganic phosphate and ADP. Three different tautomeric states were considered (Figure S7). From the ATP hydrolysis products obtained above, one of the protons of the inorganic phosphate can be transferred to ADP, resulting in the formation of hydrogen phosphate (HPO_4_^2–^) and ADP^2–^. According to the calculated B3LYPD3/MM free energy profile (shown in Figure S7), this proton transfer has a free energy barrier of 10.4 kcal·mol^–1^, while the new products are 10.5 kcal·mol^–1^ more stable. The proton can be transferred back to one of the oxygen atoms of the inorganic phosphate that is not coordinated to the Mg^2+^ ion. This strengthens their interaction and results in a more stable pose of H_2_PO_4_^–^ in the active site, 8.3 kcal·mol^–1^ below that of the products of ATP hydrolysis.

**Figure 5:**
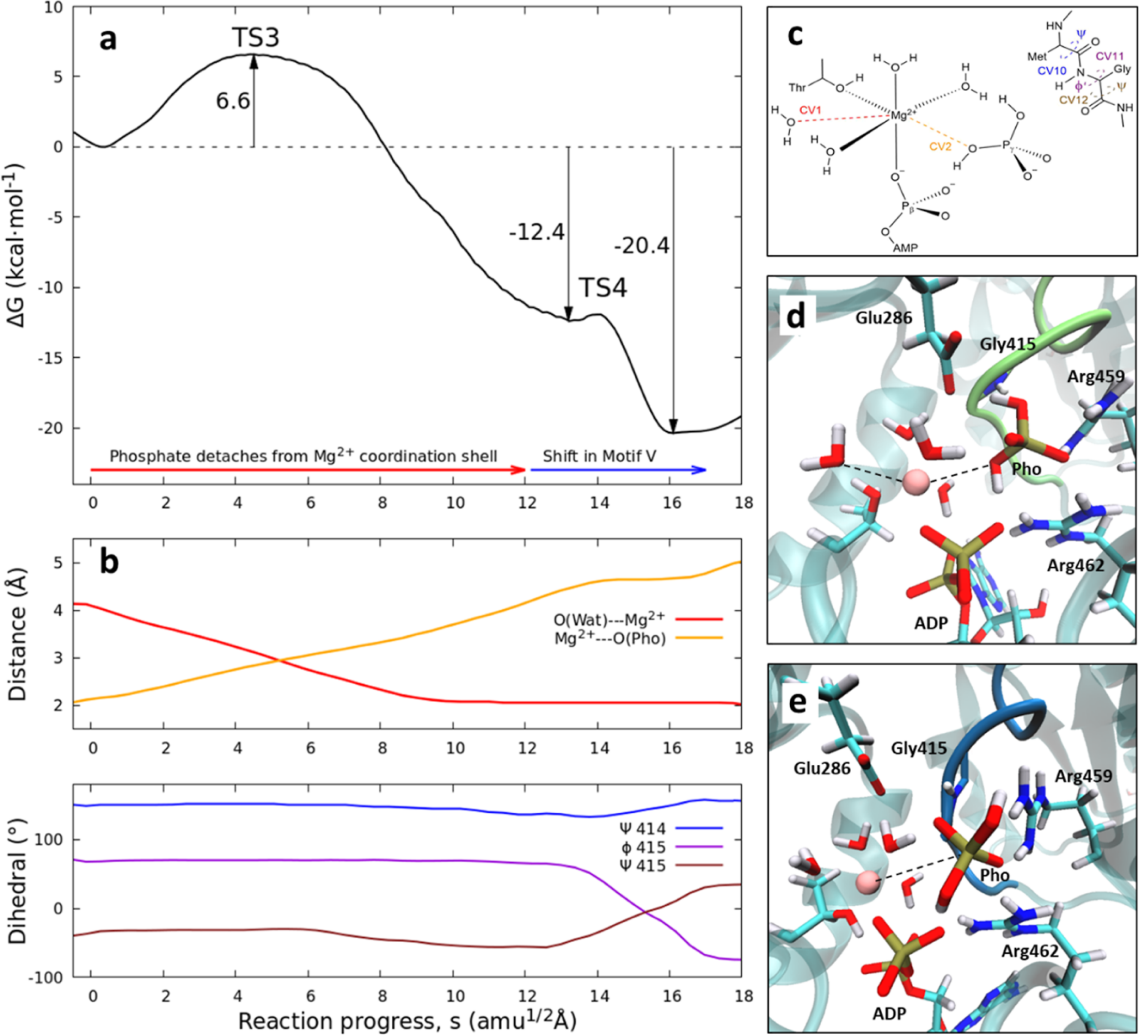
Inorganic phosphate detachment from Mg^2+^ followed by a conformational change in motif V. (a) MM free energy profile along the path-CV (s). (b) Evolution of the main CVs that define the process along the path-CV. (c) Definition of the main CVs used to study the detachment process (see Figure S8 for a complete description). (d) Structure of TS3. (e) Structure of TS4.

Alternatively, there is another mechanism for the release of free energy after ATP hydrolysis that is more favorable from the kinetic and thermodynamic points of view and that is also more relevant for the functioning of the helicase as a biological motor. According to our MD simulations, the inorganic phosphate produced by the ATP hydrolysis can detach from the Mg^2+^ ion. The vacant place in the coordination shell is then taken by a water molecule and the phosphate is displaced toward a narrow channel lined by residues Pro195, Ala316, Thr317, Pro326, Ala455, and Gln456. This has been proposed to be the exit channel for the phosphate group based on an X-ray structure of the Dengue virus NS3 helicase (PDB code: 2JLY).^48^ Interestingly, the detachment of dihydrogen phosphate during the simulation is followed by a conformational change in motif V, from conformation A to conformation C. To elucidate this process, the MFEP corresponding to the detachment of dihydrogen phosphate from the coordination shell of the divalent cation and the conformational change in motif V were explored by the ASM using MM simulations. The resulting free energy profile and converged mechanism are shown in Figures 5 and S8. The detachment of inorganic phosphate decreases the free energy of the system by 20.4 kcal·mol^–1^, which renders the whole detachment process clearly exergonic in two steps. The first step consists in the exchange of inorganic phosphate with a water molecule in the coordination shell of the Mg^2+^ ion and the displacement of the inorganic phosphate toward the exit channel. This process reveals an energy barrier of only 6.6 kcal·mol^–1^, smaller than that for the proton tautomerism. The transition state (TS3) is shown in Figure 5d, where the O(Wat)–Mg^2+^ and the Mg^2+^–H_2_PO_4_^–^ distances are both 3.0 Å (see CV1 and CV2 in Figure 5b). After TS3, the free energy decreases to −12.4 kcal·mol^–1^ when the phosphate is completely detached from the Mg^2+^ ion, reaching a distance of 4.5 Å. The driving force for this part of the process is the electrostatic repulsion between H_2_PO4^–^ and ADP^3–^, which decreases as the distance increases. During the second step, the conformational shift in motif V occurs, changing from conformation A to conformation C. This process takes place with a very small free energy barrier. Figure 5e displays the associated TS4 structure, where the phosphate group is already detached from the Mg^2+^ ion and motif V is in an intermediate conformation between A and C, as reflected by the orientation of the backbone NH group of Gly415. The free energy released during this conformational change is −8.0 kcal·mol^–1^. This energy release is led by the formation of a hydrogen bond between the Gly415 backbone NH group and the side chain of Glu286. Thus, the conformational landscape associated with motif V dramatically changes after the detachment of inorganic phosphate from the coordination shell of the divalent ion. While in the ATP-bound state, the free energy change associated with the conformational change from A to C is 4.4 kcal·mol^–1^, and once the γ-phosphate group has been moved out of the active site, the same conformational change becomes spontaneous. The backbone NH group of Gly415 acts as a sensor for the γ-phosphate group and can trigger large changes in the conformational landscape of motif V depending on the presence of this group within the active site.

Thus, the overall enzymatic ATP hydrolysis process can be seen as the combination of the ATP hydrolysis reaction, resulting in ADP and dihydrogen phosphate, and the detachment of this last group from the coordination shell of the Mg^2+^ ion. While the first step itself is endergonic by 7.6 kcal·mol^–1^, detachment diminishes the free energy by 20.4 kcal·mol^–1^. The complete enzymatic hydrolysis process is then clearly favorable.

## 4 Discussion

### 4.1 Helicase as a Ratchet Motor

**Figure 6:**
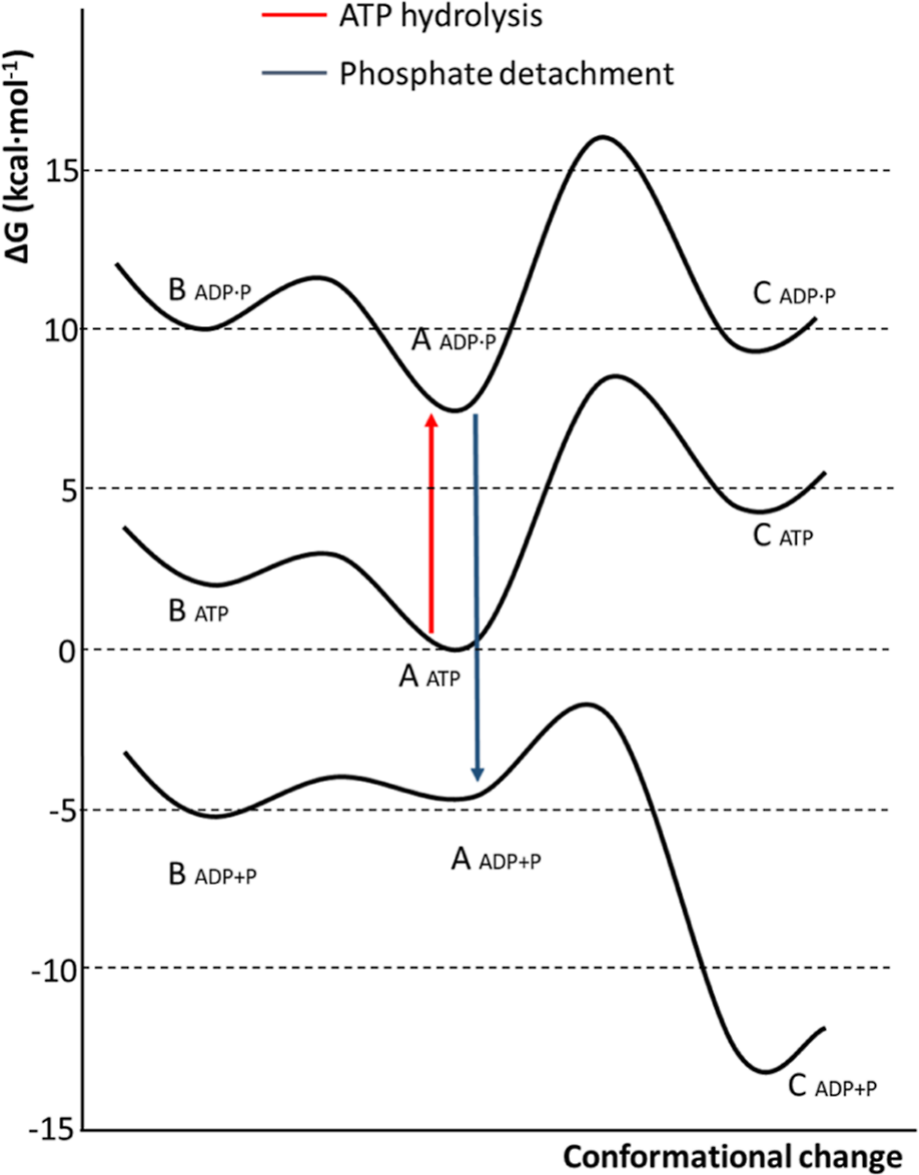
Free energy conformational landscape of motif V of the ZIKV-NS3 helicase (black curves) at different steps of the ATPase cycle. Each conformation is named X_*y*_, where X is the conformation of motif V (B, A, and C) and *y* is the ligation state (ATP, ADP·P, and ADP + P). ADP·P corresponds to the products of ATP hydrolysis, where inorganic dihydrogen phosphate is in the coordination shell of the magnesium ion. ADP + P corresponds to the state where the inorganic phosphate has been detached from the ion. The red arrow represents ATP hydrolysis (ATP → ADP·P, see Figure 4), while the blue arrow represents the detachment of inorganic phosphate (ADP·P → ADP + P, see Figure 5).

Our findings suggest that the Zika NS3 helicase operates as a ratchet motor, where its function hinges on modification of the conformational landscape of motif V, which is coupled to the ATP hydrolysis cycle. Motif V, which bridges the active sites for ATP hydrolysis and nucleic acid binding, undergoes significant conformational shifts depending on the presence of the γ-phosphate group in the active site, as summarized in Figure 6. The three black free energy curves presented in this figure correspond to the conformational landscape of motif V at different states of ATP hydrolysis: reactants (ATP), products of ATP hydrolysis (ADP and dihydrogen phosphate in contact with the magnesium ion: ADP·P), and the final products obtained after phosphate detachment (ADP + P). The arrows indicate the magnitude of the free energy change between one ligation state and the other. The red arrow, representing ATP hydrolysis (ATP → ADP·P, see Figure 4), corresponds to a free energy change of 7.6 kcal·mol^–1^, while the blue arrow, representing the detachment of inorganic phosphate (ADP·P → ADP + P, see Figure 5), shows a free energy change of −20.4 kcal·mol^–1^. The free energy curves displayed in Figure 6 show that the conformational change of motif V is spontaneous only after the motion of the phosphate group out of the active site, indicating that the absence or presence of this group determines the conformational preference of motif V.

Our simulations indicate that ATP hydrolysis predominantly occurs with motif V in conformation A (A_ATP_ in Figure 6). A hydrogen bond between the backbone NH group of Gly415 and the nucleophilic water molecule stabilizes conformation A and facilitates the hydrolysis. Following ATP hydrolysis, the resultant state A_ADP·P_ (where the phosphate is still coordinated to the metal) is anchored by the hydrogen bond formed between the NH group of Gly415 and the inorganic phosphate’s hydroxyl group. The free energy associated with ATP hydrolysis can be subsequently released as the phosphate anion moves away from ADP and leaves the metal center, resulting in the formation of A_ADP+P_, which, after overcoming a minor energy barrier, transitions spontaneously to conformation C_ADP+P_. The conformational shift is triggered by the loss of the hydrogen bond between the NH group of Gly415 and the inorganic phosphate and the formation of a new hydrogen bond with the Glu286 side chain.

The core insight is that ATP hydrolysis does not directly exert any force on motif V to provoke a conformational change. Rather, the presence or absence of the γ-phosphate group adjusts motif V’s equilibrium conformational landscape, prompting its transformation. The backbone NH group of Gly415 acts as an γ-phosphate sensor through hydrogen bond formation with different acceptors. This conformational adaptation serves as a bridge between hydrolysis and helicase functions. As per our model, motif V’s conformational change, which is nestled between the ATP and RNA binding sites, could lead to a modification in the interaction between the protein and RNA. In essence, our simulations provide an accurate microscopic view into the Zika helicase’s operation as a motor.

The description obtained from our simulations for the coupling between ATPase and helicase activities is also probably valid for other ATP-based biomolecular motors. Regarding ATP hydrolysis, formation of dihydrogen phosphate seems to be an important requisite to facilitate the release of the phosphate group from the active site because hydrogen phosphate could remain tightly bound to the magnesium ion. The spontaneity of the enzymatic ATP hydrolysis to form ADP and dihydrogen phosphate would be driven by the electrostatic repulsion between the two fragments (ADP and inorganic phosphate), which leads to the detachment of the phosphate group. This process is relevant for the functioning of the biomolecular motor because the presence/absence of the γ-phosphate group promotes a conformational change of some loops in the enzyme, favoring its displacement. The existence of a γ-phosphate sensor mechanism could be a general feature of biomolecular motors and has been proposed to be in action also for myosin.^49^ The formation or breaking of interactions with the inorganic phosphate could act as a trigger for conformational changes in those loops surrounding the ATPase site, modifying the interplay of the enzyme with the nucleic acid. In the case of the SF2 helicase superfamily, the mechanism deduced from our simulations is supported by the observation of conformational changes in motif V, not only for ZIKV^14^ but also in the case of the NS3 helicase from HCV, for which significant conformational changes are observed in the same conserved motif after binding a substrate analogue.^50^ These findings stress the importance of searching for conformational diversity in the structures of biomolecular motors in different ligation states. In particular, the development of adequate analogues for different stages of ATP hydrolysis (reactants, products, detached products, and TS), in combination with atomistic simulations, could pave the way for a complete understanding of the functioning of ATP-based biomolecular motors.

## 5 Conclusions

In this research, both MM and QM/MM simulations were employed to probe the ATP hydrolysis reaction in the Zika NS3 helicase’s active site. This enzyme acts as a biological motor, with ATP hydrolysis facilitating its translocation along the nucleic acid strand. We show that changes in the conformational landscape of motif V, a conserved motif linking the ATPase active site and the nucleic acid binding site, might underlie the function of this helicase as a biomolecular motor.

Our B3LYPD3/MM simulations revealed that the hydrolysis process occurs through a base-assisted mechanism. It starts with the separation of the γ-phosphate group from ADP to form a planar meta-phosphate group. The process continues with the nucleophilic attack of a water molecule, concerted with its deprotonation by Glu286. In the last step, the proton is transferred from Glu286 to the inorganic phosphate to form H_2_PO_4_^–^ coordinated to the Mg^2+^ ion. The obtained activation free energy is 15.1 kcal·mol^–1^, in good agreement with the experimental estimation, 17.3 kcal·mol^–1^. The reaction free energy at this point is +7.6 kcal·mol^–1^. Then, free energy can be released after proton transfer from inorganic phosphate to ADP. However, the preferred path for the relaxation of the products, both kinetically and thermodynamically, involves the detachment of the phosphate group from the coordination shell of the Mg^2+^ ion. Importantly, this process leads to a spontaneous conformational change of motif V.

Our findings clarify the atomistic details of the functioning of Zika NS3 helicase as a biological motor. The conformational transitions of motif V during the ATPase cycle are primarily guided by Gly415’s backbone NH group. This entity acts as a molecular sensor for the γ-phosphate group in the active site, establishing hydrogen bond interactions that can favor different conformations of motif V. These conformational changes could foster variations in the enzyme’s binding strength to the nucleic acid, enabling the enzyme’s translocation along the strand and, thus, its helicase action. Further simulations with RNA bound to the helicase are needed to unveil this biological motor’s complete operational cycle. Our conclusions, both on the nature of the ATP hydrolysis mechanism and on the conformational changes associated with the γ-phosphate group, could be valid to explain the origin of motion in other biomolecular motors.

## Data Availability

Raw data, parameters, coordinates, and input files for the simulations performed in this study are available at https://github.com/emedio/ZikaNS3helicase, while examples of MM and QM/MM trajectories are found in https://zenodo.org/record/8386974.

## Supporting Information Available

Table of dihedral angles in X-ray structures; probability distribution of torsional angles of Motif V; DFTB3/MM results for ATP hydrolysis in the ZIKv-NS3 helicase in conformation A of Motif V; B3LYP/MM results for the base-assisted mechanism for ATP hydrolysis in the ZIKV-NS3 helicase in conformation B of Motif V; DFTB3/MM results for ATP hydrolysis in the ZIKv-NS3 helicase in conformation B of Motif V; B3LYP/MM results for the substrate-assisted mechanism for ATP hydrolysis in the ZIKV-NS3 helicase in conformation A of Motif V; B3LYP/MM results for the proton tautomerism for ATP-hydrolysis products in the ZIKV-NS3 helicase; evolution of the full set of CVs employed to study the detachment of inorganic phosphate followed by a conformational change in Motif V; and definition of the QM region (PDF)
