# Supplementary material for: Conformational Changes and ATP Hydrolysis in Zika Helicase: The Molecular Basis of a Biomolecular Motor Unveiled by Multiscale Simulations
Source: J Am Chem Soc. 2023 Nov 3;145(45):24809–19. doi: 10.1021/jacs.3c09015 (PMC10852352; doi:10.1021/jacs.3c09015)
Supplement: Supplementary file 1 — ja3c09015_si_001.pdf [file ja3c09015_si_001.pdf]

Supplementary Information

Conformational Changes and ATP Hydrolysis in Zika Helicase. The Molecular Basis of a Biomolecular Motor Unveiled by Multiscale Simulations

Adrián García Martínez, Kirill Zinovjev, José Javier Ruiz-Pernía*, Iñaki Tuñón*

Departamento de Química Física, Universidad de Valencia, 46100, Bujassot (Spain)

to whom correspondence should be addressed:

j.javier.ruiz@uv.es; ignacio.tunon@uv.es

Table S1. Dihedral angles of Motif V in conformations A and B of Zika NS3h in the X-ray structure (PDB: 6S0J). In bold the values of the analyzed dihedral angles.

Dihedral angle	Conformation A (°)	Conformation B (°)
Ψ_{405}	139.1	139.1
Φ_{406}	-105.4	-105.4
Ψ_{406}	117.6	117.6
Φ_{407}	-102.7	-102.7
Ψ_{407}	114.7	114.7
Φ_{408}	-137.0	-137.0
Ψ_{408}	149.4	149.4
Φ_{409}	-77.8	-77.8
Ψ_{409}	-174.0	-174.0
Φ_{410}	-66.7	-66.7
Ψ_{410}	-10.3	-10.3
Φ_{411}	-61.3	-61.3
Ψ_{411}	-35.7	-22.6
Φ_{412}	-74.1	-76.2
Ψ_{412}	-9.8	-5.3
Φ_{413}	-78.0	-72.5
Ψ_{413}	-16.3	-6.1
Φ_{414}	-131.0	-106.0
Ψ_{414}	**145.1**	**57.6**
Φ_{415}	**65.1**	**92.1**
Ψ_{415}	-3.4	21.1
Φ_{416}	-83.9	-78.9
Ψ_{416}	128.2	130.2
Φ_{417}	-104.3	-104.3

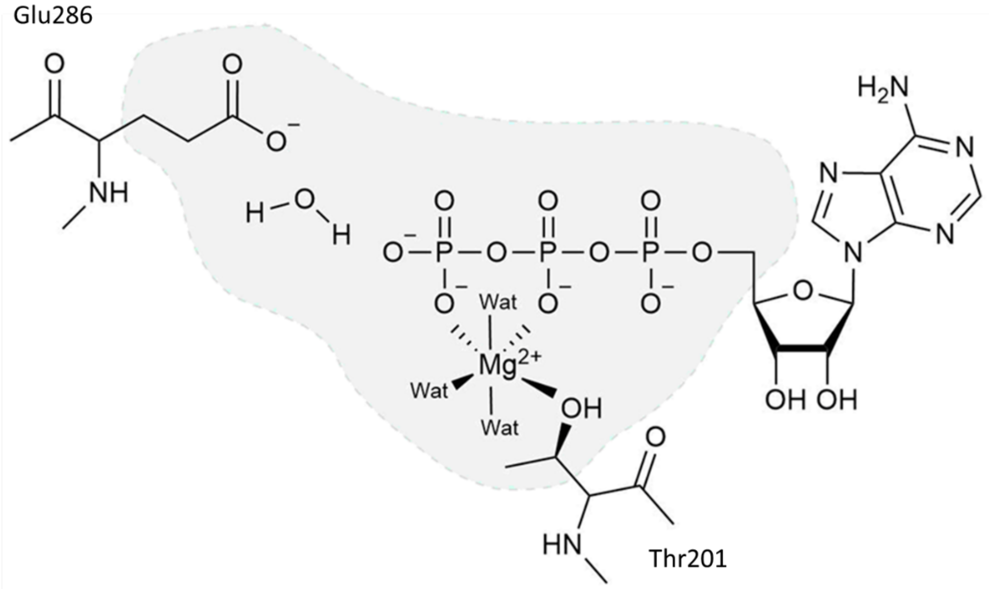

Figure S1. QM region selected to study the ATP hydrolysis reaction. The atoms included in the QM region are located inside the grey area.

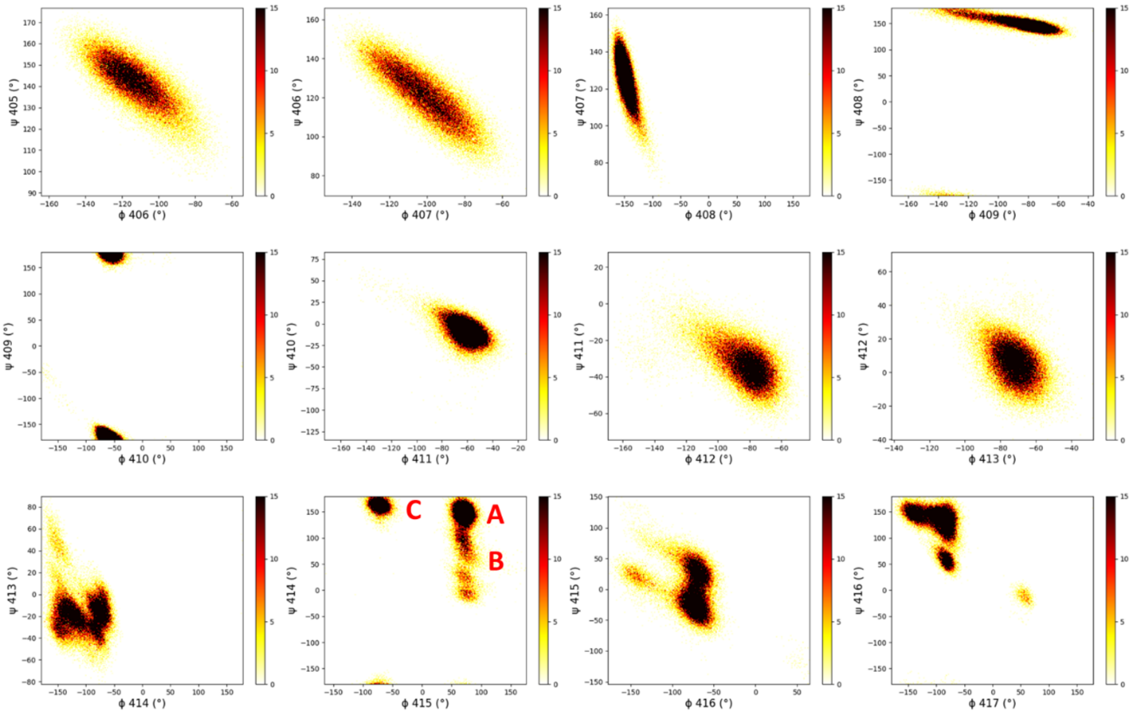

Figure S2. Configurational space of Motif V during reactants state classical simulations of Zika NS3h. Each panel corresponds to the probability distribution of pair of Ψ_i/ Φ_{i+1} torsional angles (in °) from Ψ_{405}/ Φ_{406} to Ψ_{416}/ Φ_{417}. Colourbar represents the number of samples in each bin. The three different conformations found for the Ψ_{414}/ Φ_{415} pair are labelled as A, B and C.

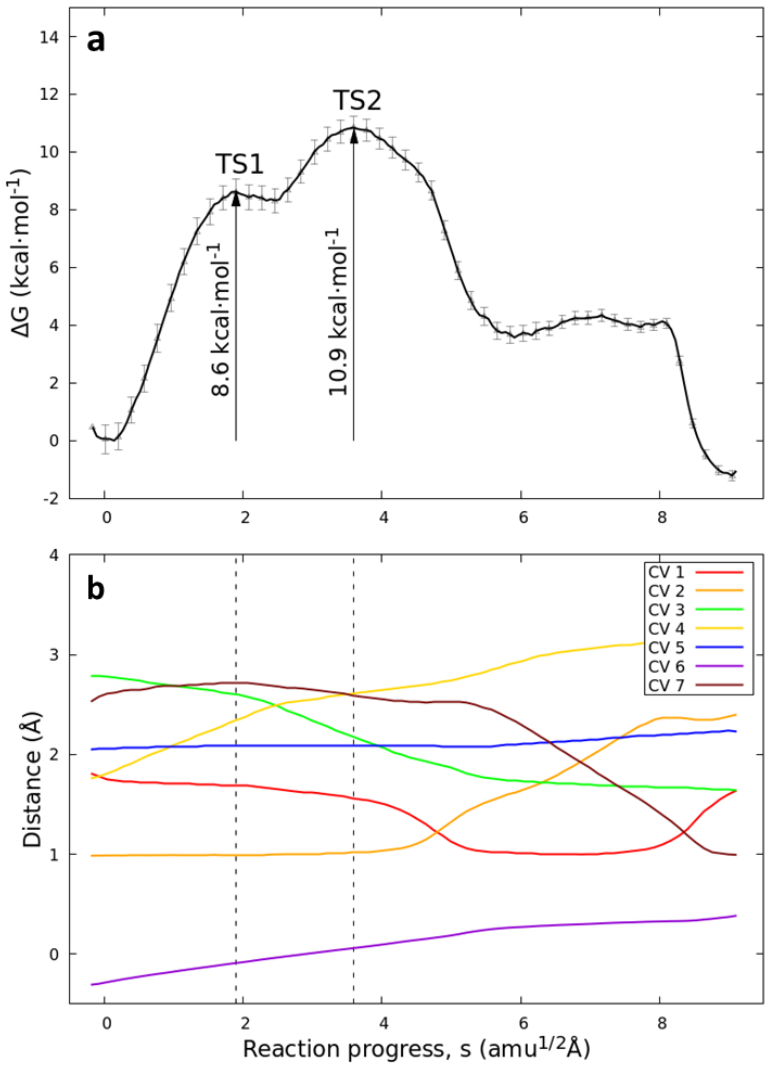

Figure S3. ATP hydrolysis in ZIKv-NS3 helicase in conformation A of motif V. **(a)** DFTB3/MM free energy profile along the path-CV (s) for ATP hydrolysis up to the formation of ADP and dihydrogen phosphate. **(b)** Evolution of the CVs along the path-CV. CVs are defined in Figure 3 of the manuscript.

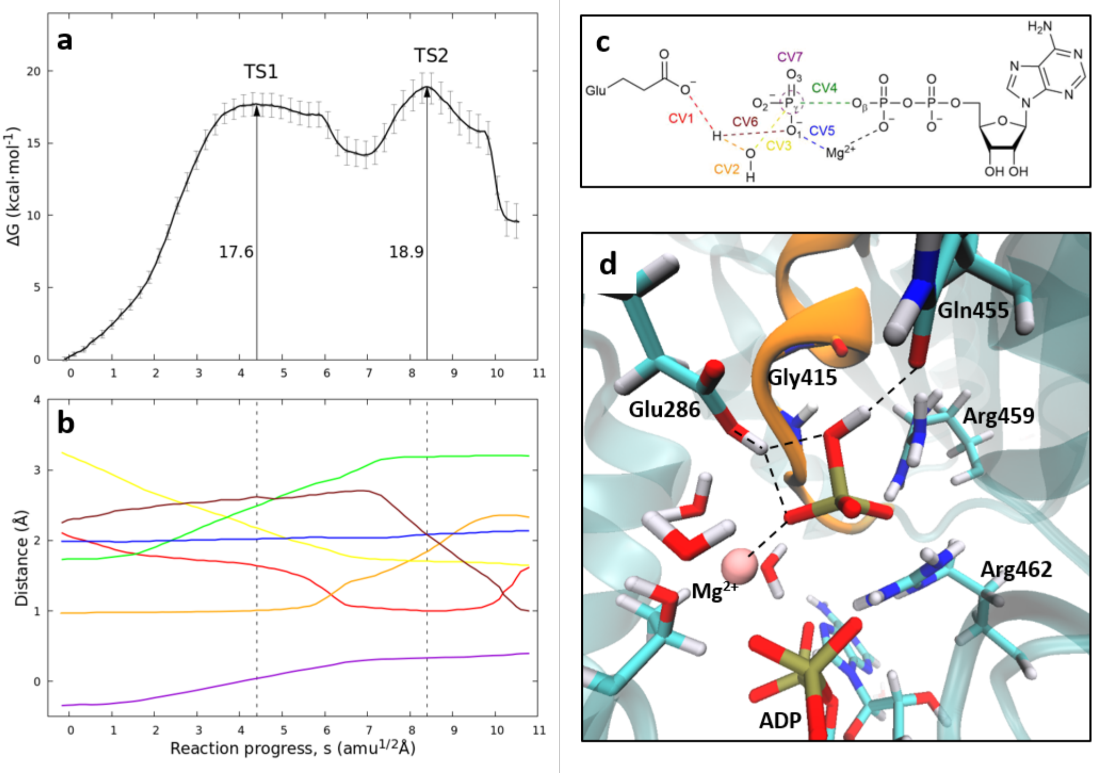

Figure S4. Base-assisted mechanism for ATP hydrolysis in ZIKV-NS3 helicase in conformation B of motif V. **(a)** B3LYPD3/6-31G*/MM free energy profile along the path-CV (s) for ATP hydrolysis up to the formation of ADP and dihydrogen phosphate. **(b)** Evolution of the CVs along the path-CV. **(c)** CVs employed to study the base-assisted reaction mechanism. **(d)** Structure of the rate-limiting step (TS2). Motif V is shown in orange colour.

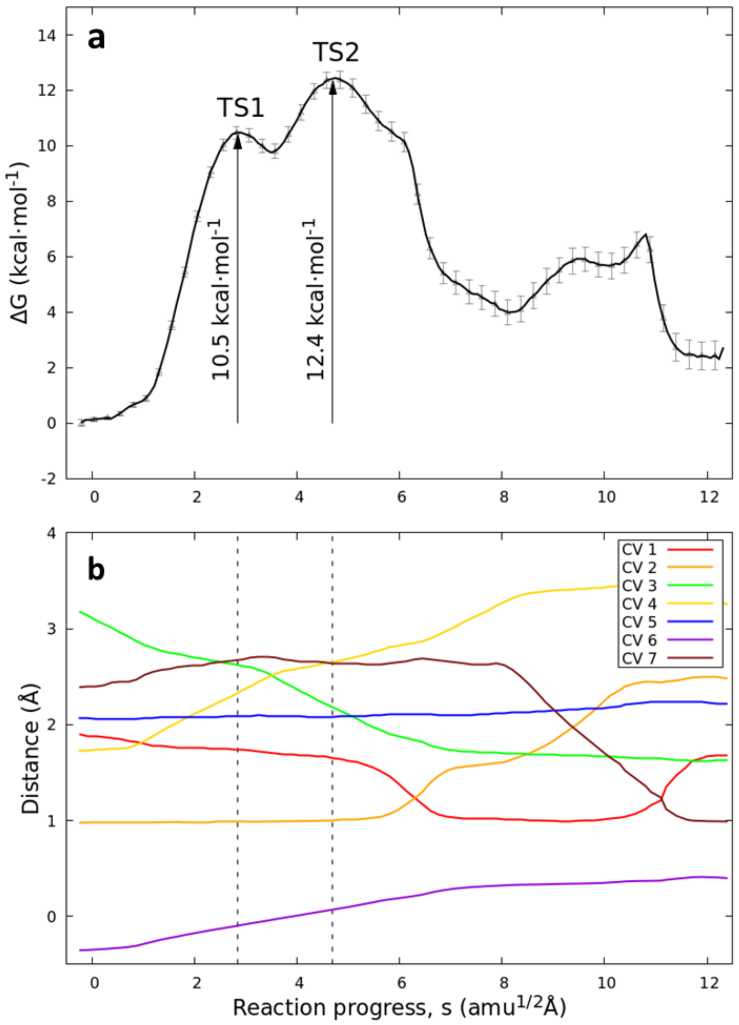

Figure S5. ATP hydrolysis in ZIKV-NS3 helicase in conformation B of motif V. **(a)** DFT3B3/MM free energy profile along the path-CV (s) for ATP hydrolysis up to the formation of ADP and dihydrogen phosphate. **(b)** Evolution of the CVs along the path-CV. The used CVs are those presented in Figure S3.

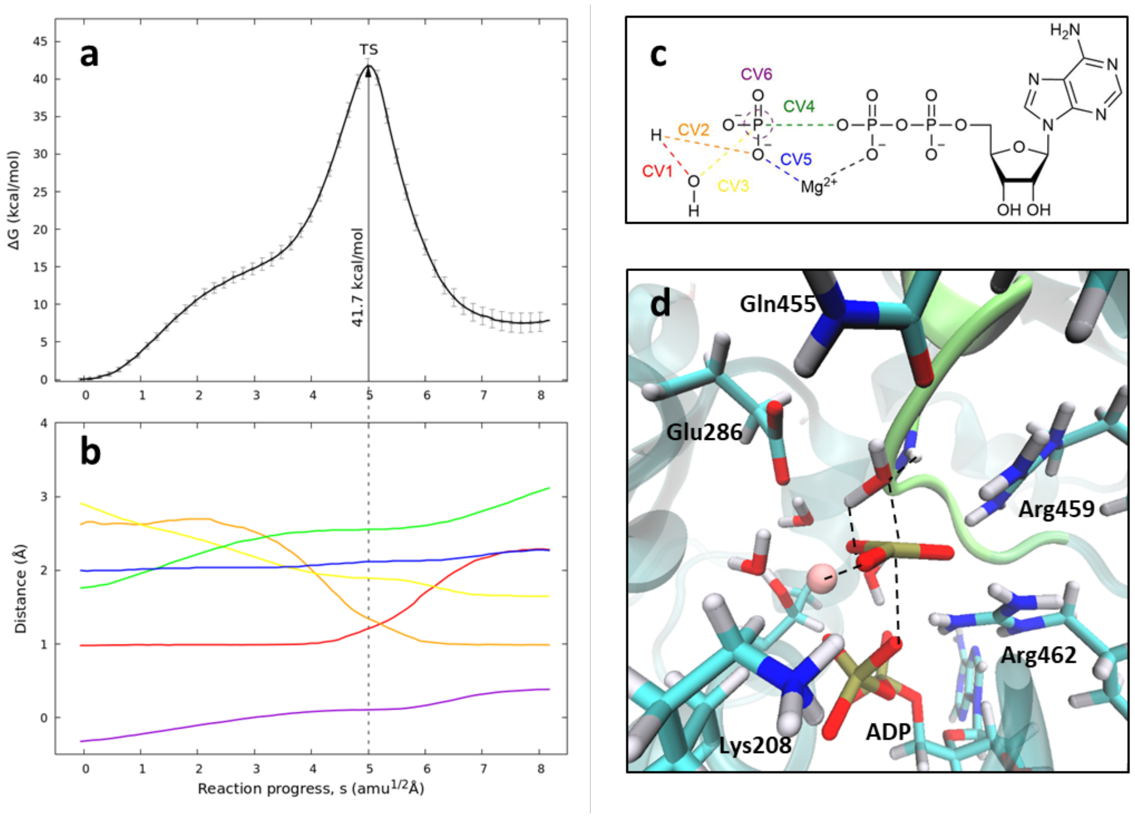

Figure S6. Substrate-assisted mechanism for the ATP hydrolysis in ZIKV-NS3 helicase in conformation A of motif V. **(a)** B3LYPD3/6-31G*/MM free energy profile along the path-CV (s) for ATP hydrolysis up to the formation of ADP and dihydrogen phosphate **(b)** Evolution of the CVs along the path-CV **(c)** CVs employed to study the substrate-assisted reaction mechanism. **(d)** Structure of the TS. Motif V is depicted in lime colour.

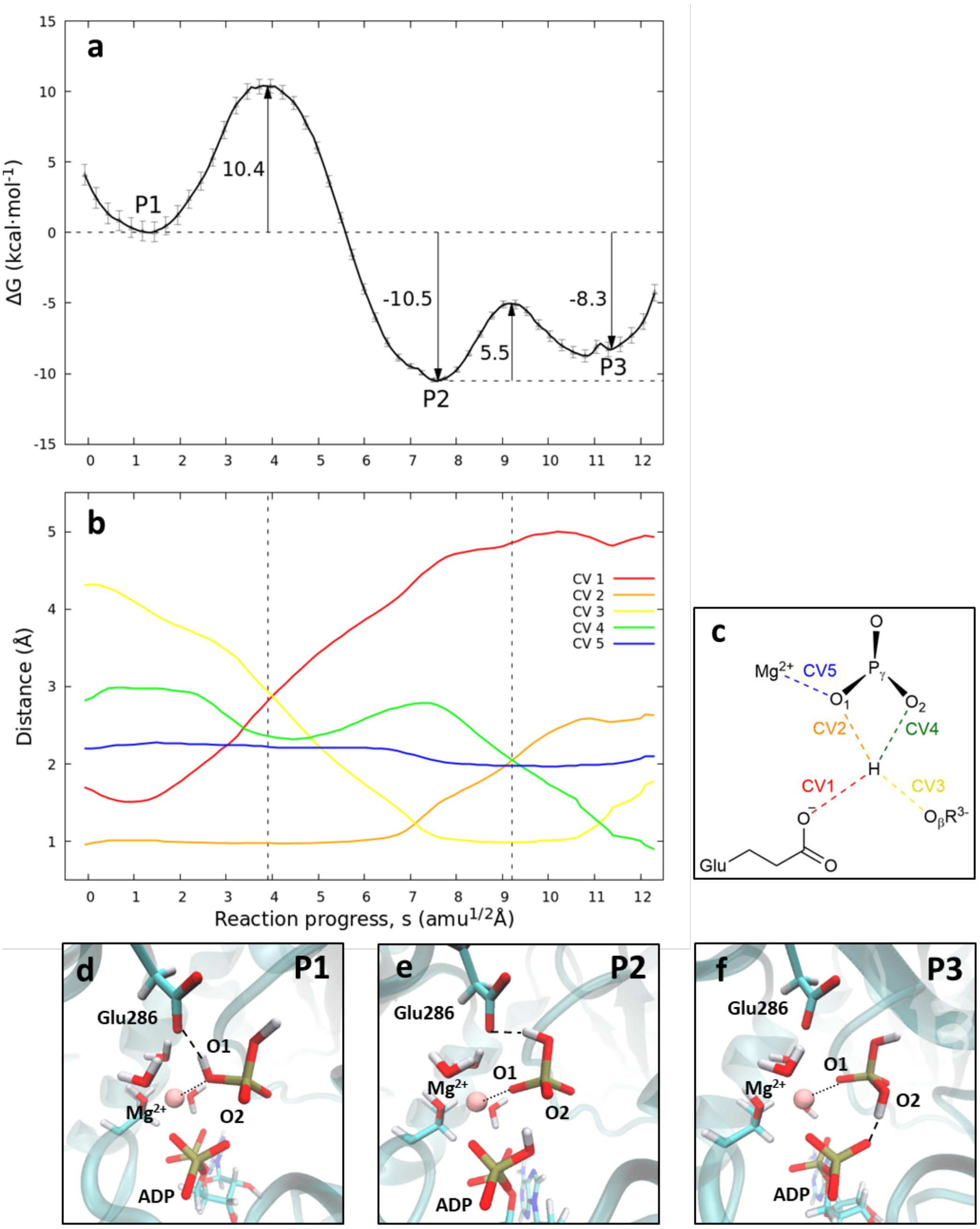

Figure S7. Proton tautomerism for ATP-hydrolysis products in ZIKV-NS3 helicase. **(a)** B3LYPD3/6-31G*/MM free energy profile along the path-CV (s) for the interconversion of P1, P2 and P3. **(b)** Evolution of the CVs along the path-CV. **(c)** CVs employed to study the double proton transfer from P1 to P2 and from this to P3. **(d)** Structure of P1, corresponding to ADP^{3-} and $H_2PO_4^-$ coordinated to the Mg^{2+} ion through a hydroxyl oxygen atom. **(e)** Structure of P2, corresponding to ADP^{2-} and HPO_4^{2-} coordinated to the Mg^{2+}. **(f)** Structure of P2, corresponding to ADP^{3-} and $H_2PO_4^{2-}$ coordinated to the Mg^{2+} ion through a non protonated oxygen atom.

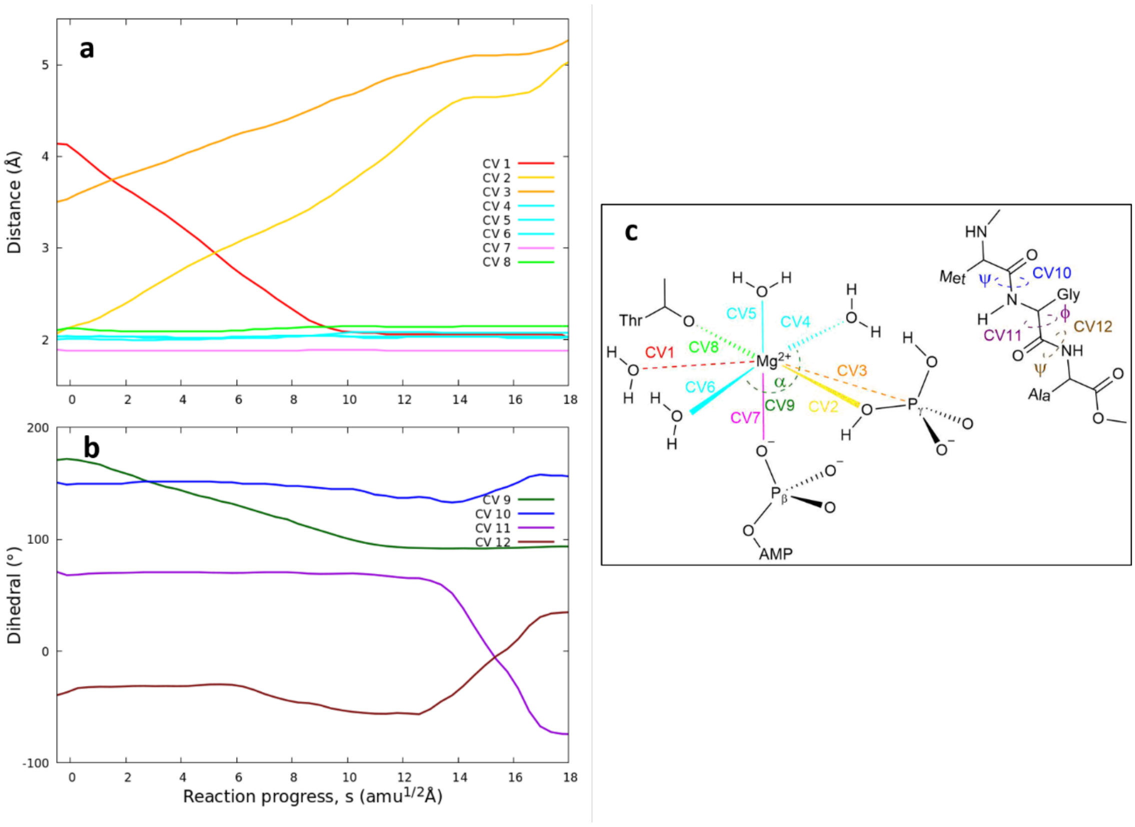

Figure S8. Evolution of the full set of Collective variables (CVs) employed to study the detachment of inorganic phosphate followed by a conformational change in Motif V. **(a)** Evolution of the distance CVs along the path-CV. **(b)** Evolution of the angle and dihedral CVs along the path-CV. **(c)** Representation of the CVs employed to study the process.

Data Availability Statement

Raw data, parameters, coordinates and input files for the simulations performed in this study are available at https://github.com/emedio/ZikaNS3helicase, while examples of MM and QM/MM trajectories are found in https://zenodo.org/record/8386974.
